# Changes in the gut microbiota diversity of brown frogs (*Rana dybowskii*) after an antibiotic bath

**DOI:** 10.1186/s12917-021-03044-z

**Published:** 2021-10-21

**Authors:** Qing Tong, Li-Yong Cui, Jia Bie, Xiao-Yun Han, Zong-Fu Hu, Hong-Bin Wang, Jian-Tao Zhang

**Affiliations:** 1 College of Veterinary Medicine, Northeast Agricultural University, Harbin, 150030 China; 2 Jiamusi Branch of Heilongjiang Academy of Forestry Sciences, Jiamusi, 154002 China; 3 College of Life Science, Jiamusi University, Jiamusi, 154007 China

**Keywords:** Bacterial community, Functional prediction, Antimicrobial bath, Gentamicin, High-throughput sequencing

## Abstract

**Background:**

Captive amphibians frequently receive antibiotic baths to control bacterial diseases. The potential collateral effect of these antibiotics on the microbiota of frogs is largely unknown. To date, studies have mainly relied on oral administration to examine the effects of antibiotics on the gut microbiota; in contrast, little is known regarding the effects of bath-applied antibiotics on the gut microbiota. The gut microbiota compositions of the gentamicin, recovery, and control groups were compared by Illumina high-throughput sequencing, and the functional profiles were analysed using Phylogenetic Investigation of Communities by Reconstruction of Unobserved States (PICRUSt). Furthermore, the relationship between the structure and predicted functional composition of the gut microbiota was determined.

**Results:**

The alpha diversity indices were significantly reduced by the gentamicin bath, illustrating that this treatment significantly changed the composition of the gut microbiota. After 7 days, the gut microbiota of the recovery group was not significantly different from that of the gentamicin group. Forty-four indicator taxa were selected at the genus level, comprising 42 indicators representing the control group and 2 indicators representing the gentamicin and recovery groups. Potential pathogenic bacteria of the genera *Aeromonas*, *Citrobacter*, and *Chryseobacterium* were significantly depleted after the gentamicin bath. There was no significant positive association between the community composition and functional composition of the gut microbiota in the gentamicin or control frogs, indicating that the functional redundancy of the gut bacterial community was high.

**Conclusions:**

Gentamicin significantly changed the structure of the gut microbiota of *R. dybowskii*, and the gut microbiota exhibited weak resilience. However, the gentamicin bath did not change the functional composition of the gut microbiota of *R. dybowskii*, and there was no significant correlation between the structural composition and the functional composition of the gut microbiota.

**Supplementary Information:**

The online version contains supplementary material available at 10.1186/s12917-021-03044-z.

## Background

The diversity of bacterial communities, especially the animal gut microbiota, is closely related to animal health [1, 2]. The gut bacterial community is dynamic, continually changing in composition to adapt to changes in the internal and external environments [3]. The intestinal microbiota may form a barrier to pathogens, producing many of the required products and playing a role in digestion, intestinal morphology, immunity, and regulation of host immune gene expression [4]. With the development of high-throughput sequencing technology, microbial diversity has attracted increasing attention [2]. Currently, the body of research on the gut bacterial community of farmed animals is rapidly growing, reflecting the increasingly recognized importance of the role of the intestinal bacterial community in animal health [5]; however, research investigating aquatic products, especially amphibians, has not attracted much attention.

Antibiotics are widely used in aquaculture but are associated with several adverse side effects related to perturbation of the microbiota [6]. Various antibiotics have been used to prevent such outbreaks in aquaculture sectors. Antibiotics may be used to treat gastrointestinal infections in amphibians; however, other diseases, including bacterial diseases (such as red-leg syndrome), diseases related to improper husbandry (such as sunburn), and protozoan parasites (such as sporozoans), may also be treated with antibiotics [7]. Many studies have shown that the use of antibiotics has a significant impact on the animal intestinal bacterial community, such as a reduction in bacterial diversity, changes in species composition, the introduction of new species, and the total eradication of existing species [8, 9]. The widespread use of antibiotics has led to the rapid emergence of drug-resistant bacteria and has severely damaged the ecological stability and species diversity of animals’ healthy microbiota [9, 10]. However, few reported studies have investigated the effects of antibiotics on the gut microbiota of amphibians.

The use of baths to administer drugs to large groups of diseased or at-risk animals is suitable as a treatment regimen and preventive measure [11–13]. Due to the large number, small size, and stress susceptibility of captive frogs, intramuscular and intravenous injection methods are difficult to achieve [12]. Some species of captive frogs (such as *Rana dybowskii*) can prey only on moving objects (such as insects), and drugs are difficult to administer orally [12]. Therefore, antibiotic baths are a good method of administration and are particularly important for the treatment of frog diseases [11]. The stratum corneum (SC), considered the primary barrier to percutaneous absorption in mammals, is much thinner in frogs [11]. Thus, it is unsurprising that the limited comparative studies of chemical absorption through frog and mammalian skin have reported much higher absorption through frog skin [14]. Gentamicin has been widely used in veterinary medicine and has antibiotic activity (in vitro) against most bacterial genera commonly associated with septicaemia in frogs [15]. Gentamicin is better absorbed through the skin than other antibiotics and has less toxicity and fewer side effects before a therapeutic blood concentration is attained [16]. To date, many studies have been carried out regarding the influence of antibiotics on the gut microbiota [4, 8]. However, these studies mainly used oral antibiotics, and there has been very limited research attempting to understand the effects of bath-applied antibiotics on the bacterial community in the interior of the body, such as the intestines.

Functional predictions can connect the structure and function of gut microbiota and probably help to explain dysbiosis/perturbation [17, 18]. The functional redundancy of the gut microbiota may differ depending on the host species [19]. Each species of frog has a unique gut microbiota structure, but bacterial biofunctions are similar among species [20]. Functional redundancy strongly affects the consequences of change trajectories caused by differences in age, diet, and disease [21]. The gut microbiota differs significantly between normal and diseased animals (as observed, for example, in frogs and shrimp), and the community composition is significantly and positively correlated with the functional composition [18, 22]. Diseases may also be accompanied by the disappearance of functional redundancy, which is likely related to the intensity and advancement of dysbiosis [21]. Thus far, studies investigating the properties of the gut microbiota in amphibians have been scarce; in particular, few functional studies have investigated how changes in the intestinal microbial community impact microbially modulated functions.

*R. dybowskii* (the brown frog) is a major aquaculture species with medical and nutritional value in China [22]. The current stagnation of the *R. dybowskii* culture industry is mainly caused by diseases, primarily bacterial and parasitic diseases [12]. Antibiotic baths, a good method of drug administration, are particularly important for disease prevention in *R. dybowskii* [7, 11]. To better understand the effects of an antimicrobial bath on the gut microbiota of brown frogs, we treated the frogs with a gentamicin bath or an unmedicated bath, and the gut microbiota was compared 1 week after recovery. In this study, we hypothesized that (1) the gentamicin bath would affect the microbial diversity of the gut bacterial community in the treated frogs, (2) the gut microbiota would return to the pre-treatment state within 7 days after the bath, and (3) changes in the gut microbiota would result in changes in function, i.e., there is no functional redundancy among the intestinal microorganisms of *R. dybowskii*.

## Results

### Alpha diversity and the core microbiota

**Fig. 1:**
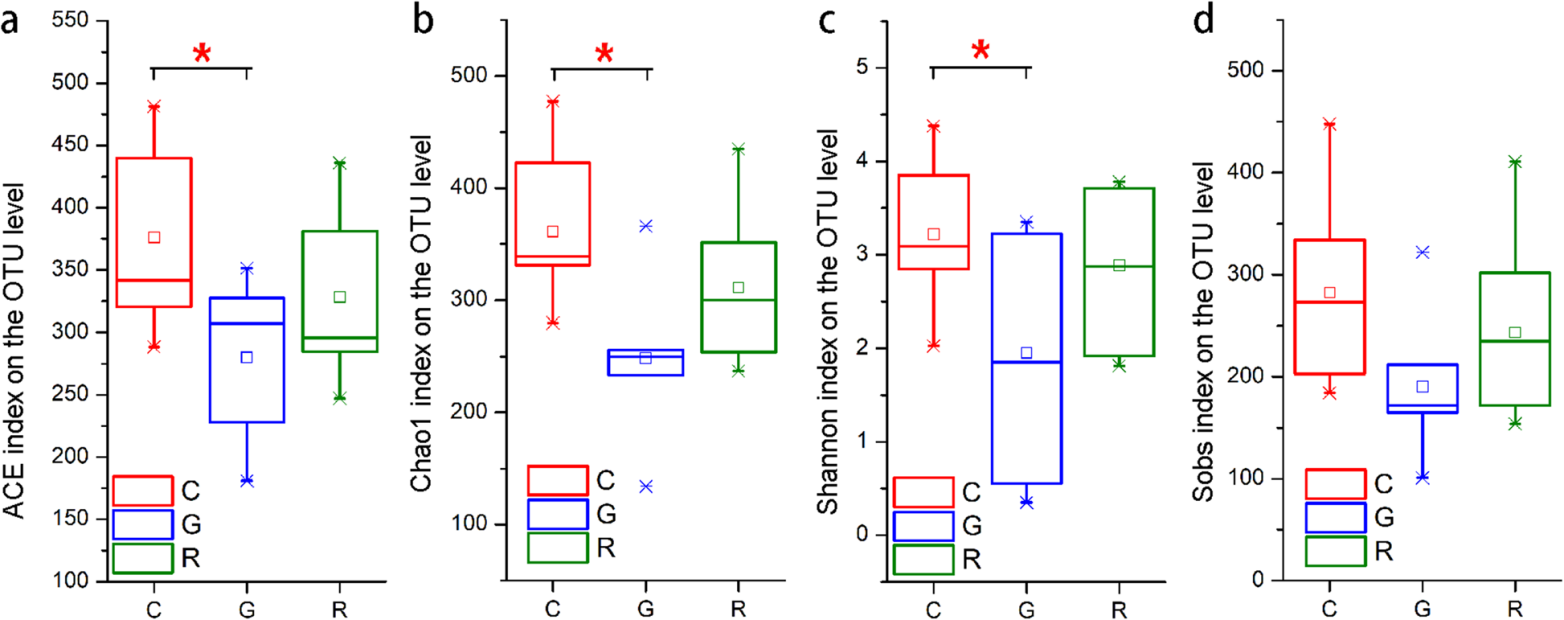
Alpha diversity of the intestinal bacterial community of *R. dybowskii. *Comparison of the alpha diversity (abundance-based coverage estimator (ACE), Chao1, Shannon and Sobs indices) of the intestinal bacterial community of *R. dybowskii *among the control (C), gentamicin (G), and recovery (R) groups (Wilcoxon rank-sum test, 0.01 <* P*≤0.05 marked as *)

The abundance-based coverage estimator (ACE), Chao1, and Shannon indices differed among the control OUT, gentamicin (G), and recovery OUT groups (Kruskal-Wallis H test; *P* < 0.05). The ACE, Chao1, and Shannon indices differed between the antibiotic bath group and the C group (Wilcoxon rank-sum test; *P* < 0.05, Fig. 1). However, there was no difference in the ACE, Chao1, Shannon, or observed richness (Sobs) index between the antibiotic bath group and the R group (Wilcoxon rank-sum test; *P* < 0.05, Fig. 1). The rarefaction curves tended to plateau, indicating that the amount of sampling was reasonable and that more sampling produced only a small number of new operational taxonomic units (OTUs) (Figure S1).

The Venn diagrams showed that the number of unique OTUs in the G group (21) was lower than that in the R group (60) or the C group (119) (Figure S2). As the number of samples increased, the number of core OTUs in the C group decreased to a lesser extent, while that in the G group decreased to a more significant extent (Figure S3a). The numbers of core OTUs in the C, G, and R groups and in all 21 frogs were 47, 25, 39, and 16, respectively (Figure S3b). The 16 core OTUs were from four phyla, including 8 from Bacteroidetes, 3 from Actinobacteria, 3 from Proteobacteria, and 2 from Firmicutes (Table S1). The most abundant core OTUs were OUT198 (*Vagococcus*), OUT179 (*Citrobacter*), OUT417 (*Bacteroides*), OUT38 (*Bacteroides*), OUT333 (*Faecalitalea*), and OUT753 (*Arthrobacter*) (Table S1).

### Gentamicin significantly changed the structure of the gut microbiota

**Fig. 2:**
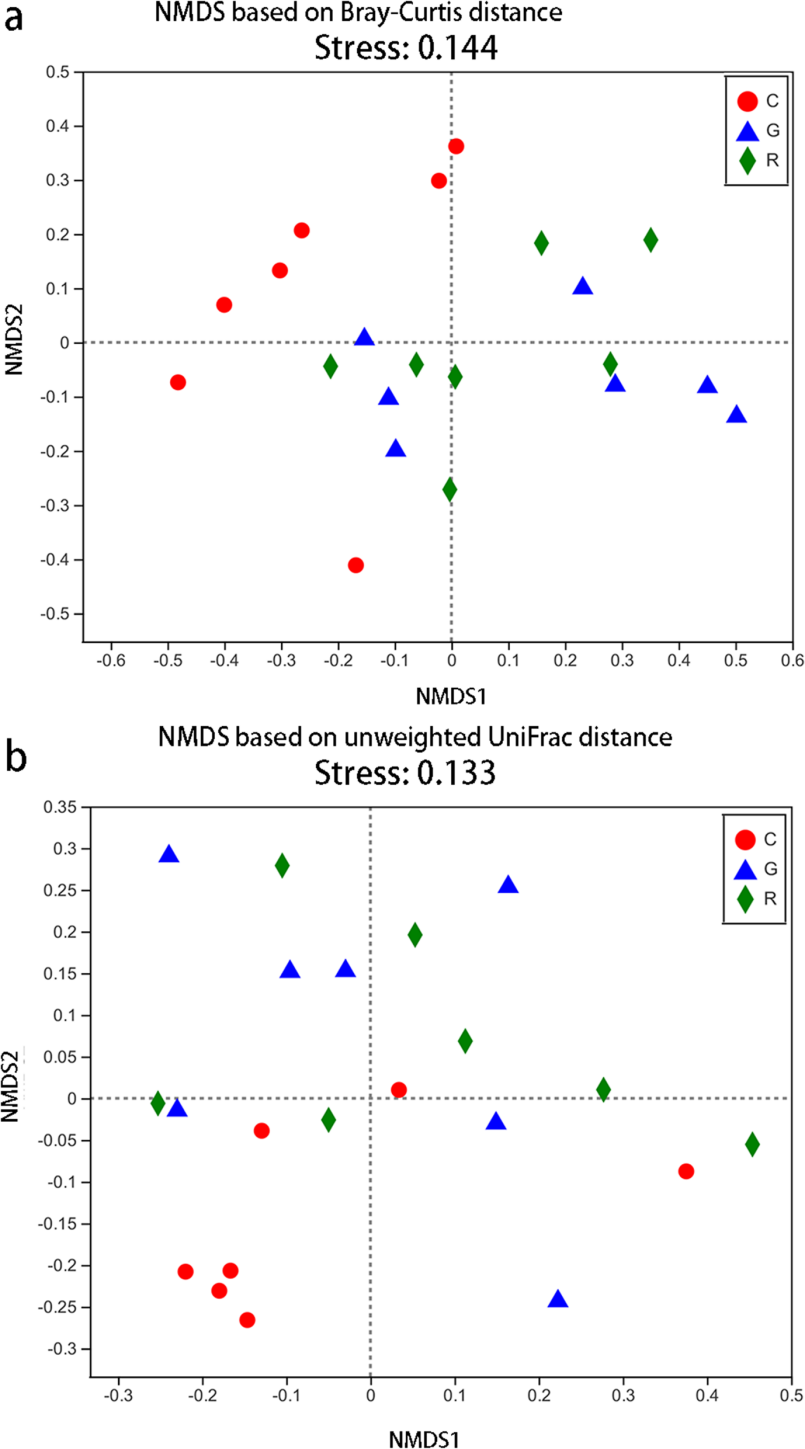
Microbial community shifts associated with an antibiotic bath in captive *R. dybowskii.* Non-metric multidimensional scaling (NMDS) shows patterns of separation in the control (red, C), gentamicin (blue, G), and recovery (green, R) groups based on Bray-Curtis (**a**) and unweighted UniFrac (**b**) distances. NMDS was based on all operational taxonomic units (OTUs). Each point represents the gut microbiota of an individual of *R. dybowskii*

The gut microbiota composition differed between the G and C groups and between the R and C groups based on the Bray-Curtis dissimilarity matrix (Adonis: *P* < 0.05; ANOSIM: *P* < 0.05; Table 1; Fig. 2) and the unweighted UniFrac distances (Adonis: *P* < 0.05; ANOSIM: *P* < 0.05; Table 1; Fig. 2). However, the gut microbiota composition did not differ significantly between the G and R groups based on the Bray-Curtis dissimilarity matrix (Adonis: *P* > 0.05; ANOSIM: *P* > 0.05; Table 1; Fig. 2) or the unweighted UniFrac distances (Adonis: *P* > 0.05; ANOSIM: *P* > 0.05; Table 1; Fig. 2). The gut microbiota was distinctly split into two major groups on the NMDS, where the C group was relatively far from the G and R groups, while the G and R groups were relatively close together (Fig. 2).

**Table 1:** Pairwise comparisons showing differences in the gut bacterial community among different groups

	Bray-Curtis				Unweighted UniFrac			
	ANOSIM		Adonis		ANOSIM		Adonis	
C vs. G	0.489	0.002	0.214	0.006	0.249	0.023	0.146	0.019
G vs. R	0.022	0.329	0.081	0.362	0.095	0.174	0.094	0.192
C vs. R	0.338	0.010	0.164	0.018	0.270	0.037	0.154	0.033
All	0.284	0.005	0.204	0.005	0.207	0.018	0.170	0.015

C indicates the control group, G indicates the gentamicin group and R indicates the recovery group

### Composition of and variation in frog gut microbiotas

**Fig. 3:**
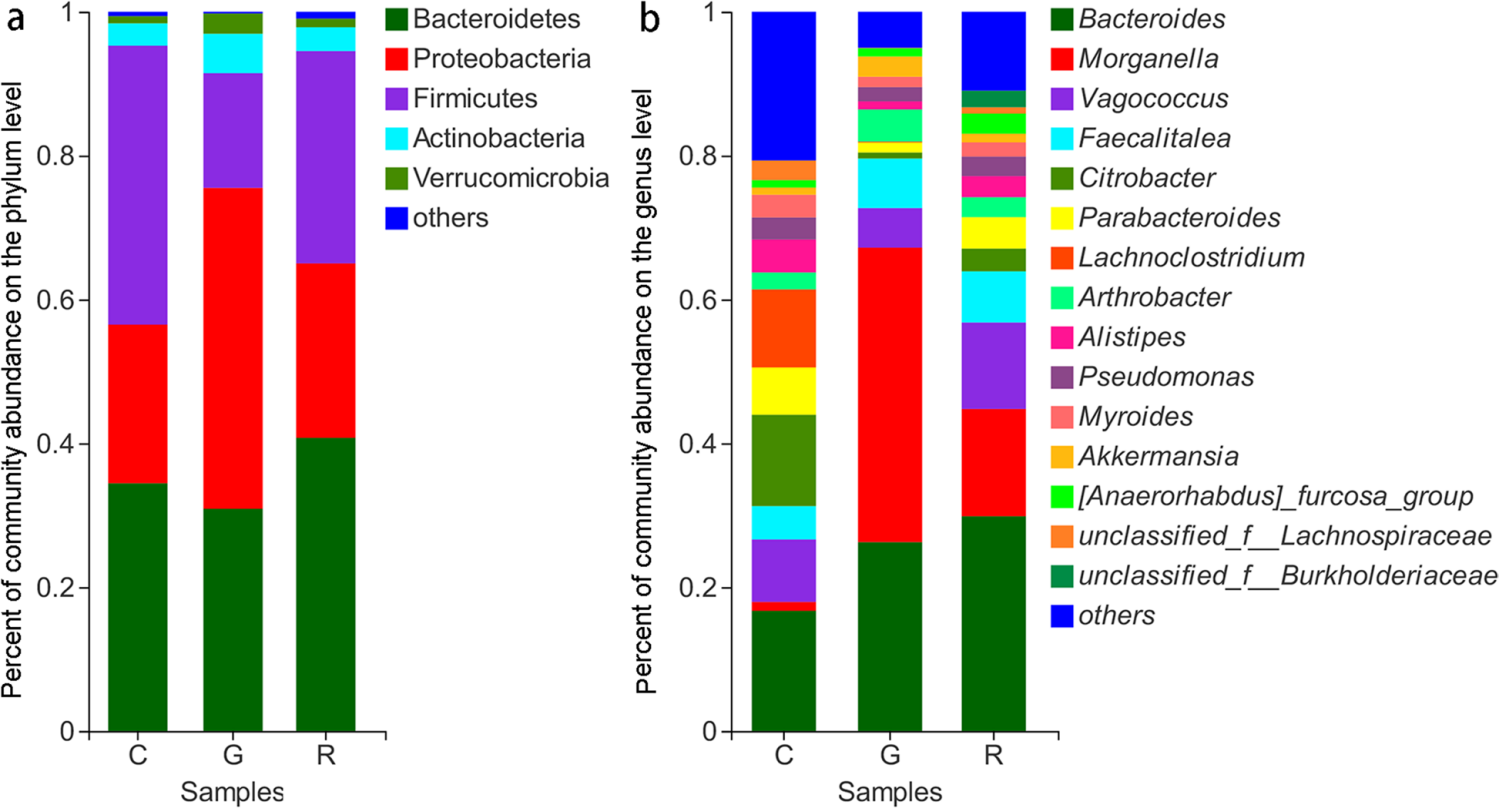
Gut microbiota composition across the groups. Gut microbiota composition across the groups at the phylum (**a**) and genus (**b**) levels. Only genera with relative abundances over 2 % in at least one sample are shown

Taxonomic assignment analysis showed that the most abundant phyla in the C, G, and R groups were Firmicutes (C: 38.19 %, G: 15.90 %, R: 30.09 %), Bacteroidetes (C: 35.15 %, G: 31.00 %, R: 40.94 %), Proteobacteria (C: 21.96 %, G: 44.74 %, R: 23.44 %), and Actinobacteria (C: 3.23 %, G: 5.37 %, R: 3.38 %) (Figs. 3a and S4). In total, 10 phyla were shared among all groups, and no bacterial phylum was significantly different among the C, G, and R groups (Kruskal–Wallis H test, FDR correction, CI: Scheffer, *P* > 0.05).

The most abundant microbial genera were *Bacteroides*, *Morganella*, *Vagococcus*, *Faecalitalea*, *Parabacteroides*, *Arthrobacter*, *Alistipes*, *Pseudomonas*, and *Myroides* (Figs. 3b and S5). Of the 290 genera, the 5 bacterial genera *Crenobacter*, *Morganella*, *unclassified_f_Eggerthellaceae*, *unclassified_f_Veillonellaceae*, and *Weissella* exhibited significant differences among the C, G, and R groups (Kruskal–Wallis H test, FDR correction, CI: Scheffer, *P* < 0.05; Figure S5).

**Fig. 4:**
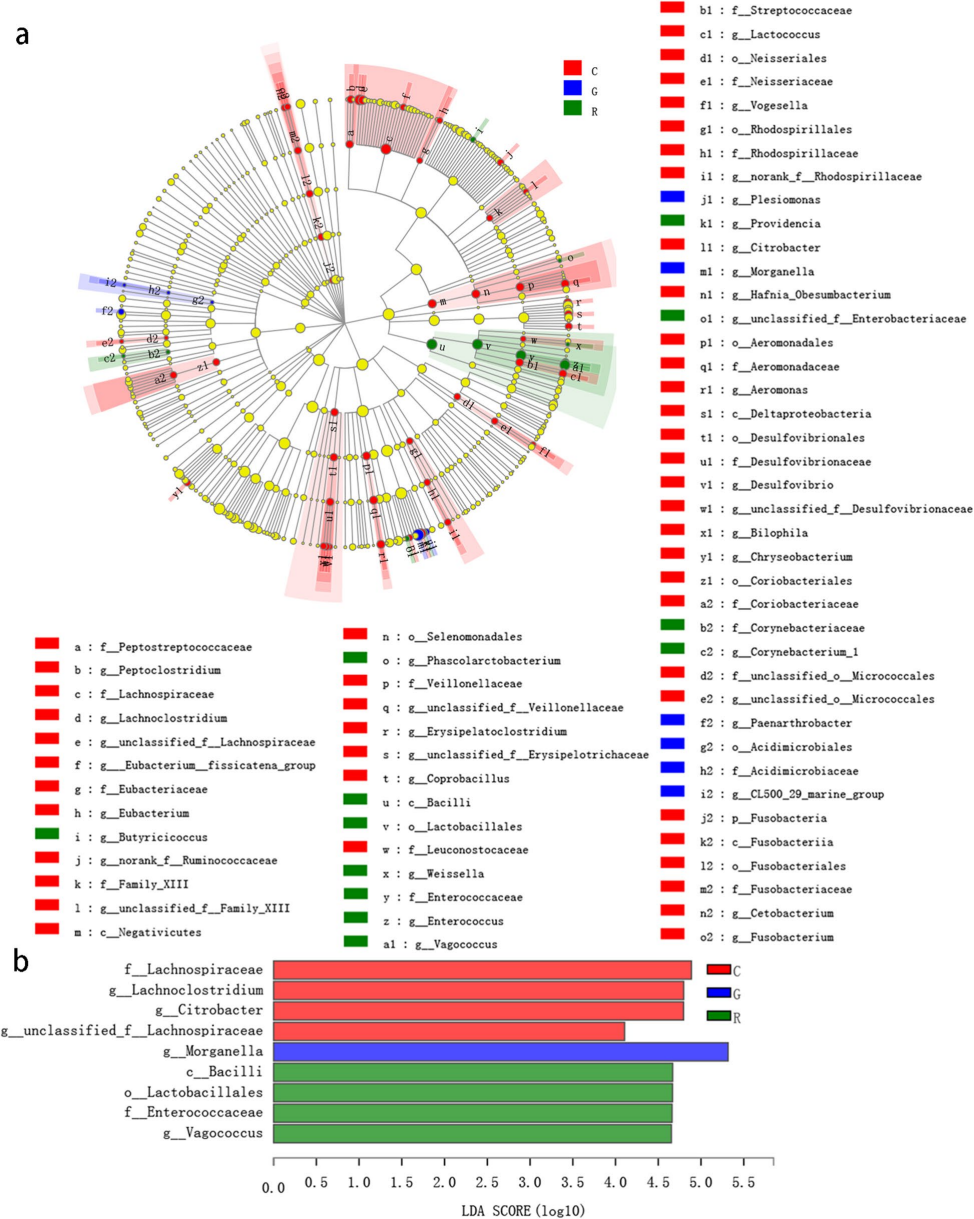
Results of linear discriminant analysis (LDA) effect size (LEfSe) analysis. Cladogram showing the phylogenetic distribution of microbial lineages connected with the differences among the control (C), gentamicin (G), and recovery (R) groups using LEfSe with LDA > 2 (**a**) or 4 (**b**). Differences among the C, G, and R groups are represented by the treatment colour (red indicates the C group, blue indicates the G group, and green indicates the R group. Each circle’s diameter is proportional to the taxon’s abundance. The multiclass analysis used an all-against-all strategy. The nested circles represent taxonomic ranks from domain to genus. Labels are shown at the class, order, and family levels

The linear discriminant analysis (LDA) effect size (LEfSe) showed that Fusobacteria were significantly enriched in the C group (LDA > 2, *P* < 0.05, Fig. 4a). Most of the bacterial taxa with significant differences were in the C group, and the G and R groups had fewer bacterial taxa (Fig. 4a). LEfSe analysis at the genus level revealed that *Morganella*, *CL500_29_marine_group*, *Paenarthrobacter*, and *Plesiomonas* were significantly enriched in the G group and that *Butyricicoccus*, *Corynebacterium_1*, *Enterococcus*, *Phascolarctobacterium*, *Providencia*, *Vagococcus*, and *Weissella* were significantly enriched in the R group (LDA > 2, *P* < 0.05, Fig. 4a). When LDA > 4, LEfSe analysis showed that at the genus level, *Citrobacter* (C group), *Morganella* (G group), and *Vagococcus* (R group) were significantly enriched (LDA > 4, *P* < 0.05, Fig. 4b).

### Indicator taxa of frog gut dysbiosis and potentially pathogenic genera

**Fig. 5:**
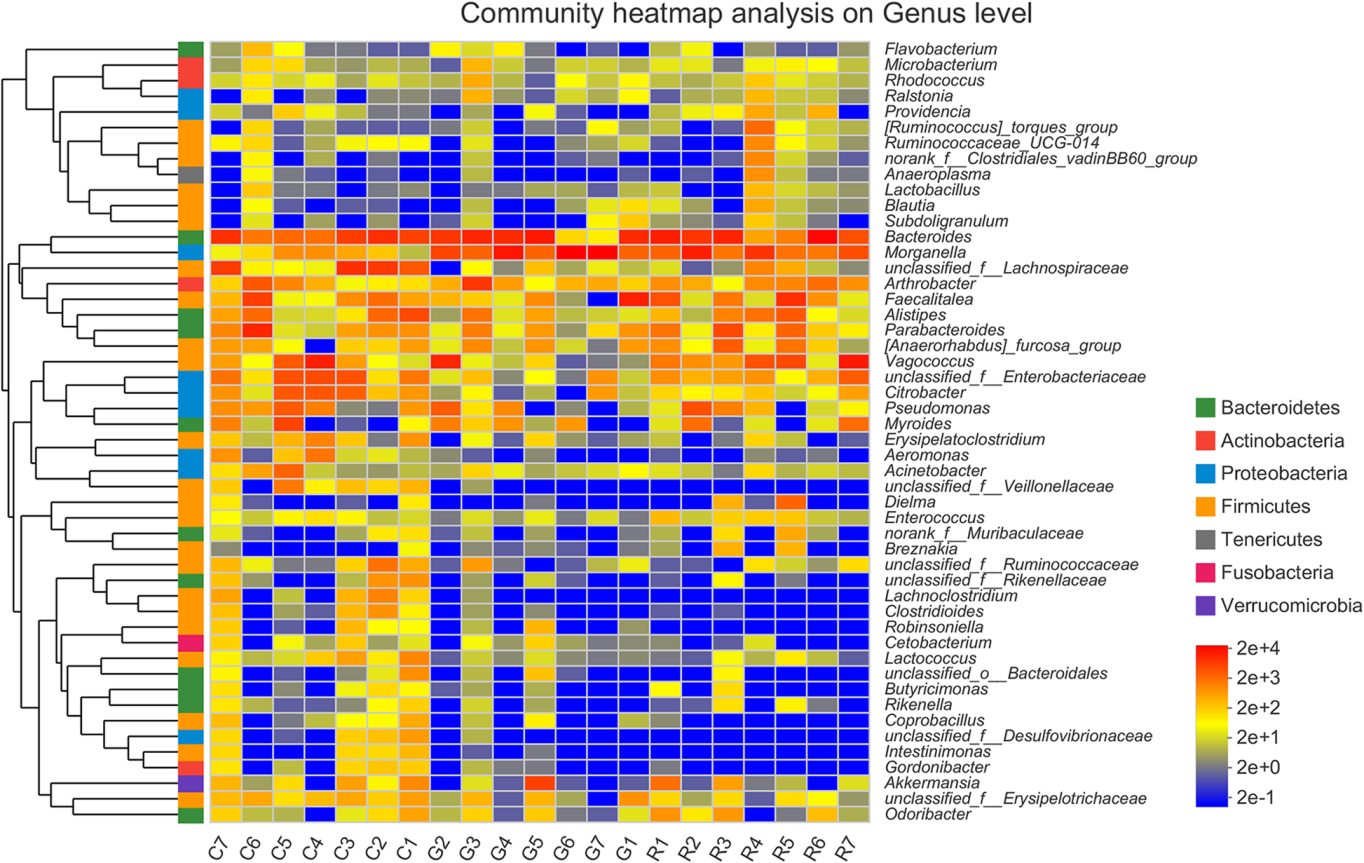
Heatmap showing the relative abundances of the indicator taxa in different groups. The labels near the ordinate and under the horizontal axis presents the names of the species and samples, respectively. The colour gradient represents the changes in abundance among the different species in the sample, and the relevant data are listed on the right side

At the genus level, forty-four indicator taxa were selected, including 42 taxa indicating the C group and 2 species (*Butyricicoccus* and *Morganella*) indicating the combined G and R groups (G+R; Fig. 5). A heatmap depicting the normalized abundances of the 44 indicator taxa across the samples was generated, showing their ability to discriminate among the samples according to the sample grouping process (Fig. 5).

The distribution and comparison of potentially pathogenic genera in the gut of the C, G, and R groups are shown in Table 2 and Figure S7. *Aeromonas*, *Acinetobacter*, and *Chryseobacterium* differed among the C, G, and R groups (Kruskal-Wallis H test; *P* < 0.05). The relative abundance of the bacterial genera belonging to *Aeromonas*, *Citrobacter*, and *Chryseobacterium* was significantly decreased in the G group (Wilcoxon rank-sum test; *P* < 0.01, Table 2). After 7 days of recovery, *Aeromonas*, *Citrobacter*, and *Chryseobacterium* still significantly differed between the C and R groups (Wilcoxon rank-sum test; *P* < 0.01, Table 2).

**Table 2:** Changes in the relative abundance of potentially pathogenic genera after antibiotic baths (Wilcoxon rank-sum test)

Genus	OTUs	C group (mean ± SD)	G group (mean ± SD)	R group (mean ± SD)
*Acinetobacter*	OUT666, OUT528, OUT332, OUT41, OUT175, OUT173 OUT160	1.51 ± 3.06^a^	0.14 ± 0.18^a^	0.09 ± 0.12^a^
*Aeromonas*	OUT2	1.25 ± 1.98^a^	0^b^	0^b^
*Citrobacter*	OUT179, OUT74	12.70 ± 11.14^a^	0.85 ± 1.71^b^	3.16 ± 3.82^b^
*Chryseobacterium*	OUT413	0.28 ± 0.41^a^	0.01 ± 0.01^b^	0^b^
*Proteus*	OUT405	0.01 ± 0.01^a^	0.01 ± 0.02^a^	0^a^
*Pseudomonas*	OUT192, OUT552, OUT185	3.06 ± 3.51^a^	1.98 ± 3.81^a^	2.71 ± 5.04^a^
*Staphylococcus*	OUT222, OUT733	0.01 ± 0.01^a^	0.02 ± 0.04^a^	0.03 ± 0.03^a^
*Streptococcus*	OUT346, OUT322	0.01 ± 0.02^a^	0.01 ± 0.0^a^	0^a^

^a,b ^significant differences (*p* < 0.05)

### Relationship between bacterial community structure and function

**Fig. 6:**
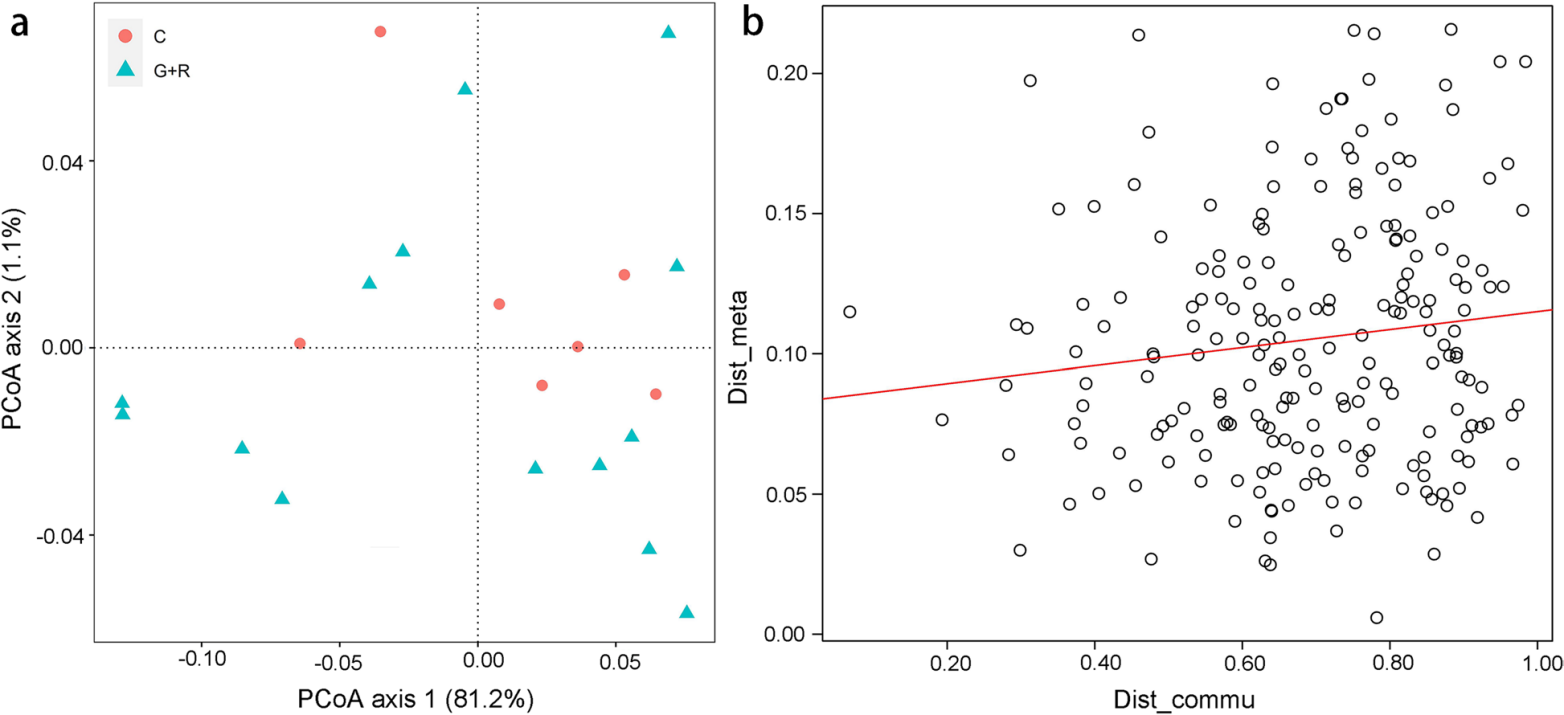
Functional differences in the gut bacterial community in different groups. Principal coordinate analysis (PCoA) of the functional features of the gut microbiota using the Bray-Curtis distance (**a**) and correlation between functional and compositional similarities (**b**)

Three hundred functional pathways were obtained in the C and G+R groups. The principal coordinate analysis did not show significant differences in the functional composition between the C and G+R groups (Fig. 6a). Similarly, the bacterial community similarity test did not show a significant difference in the functional composition between the C and G+R groups (C: G+R, ANOSIM statistic *R* = -0.041, *P* = 0.637, Fig. 6a).

The linear regression analysis showed that the gut microbiota composition and functional composition of the C and G+R groups were not significantly and positively correlated (C and G+R: r = 0.125, *P* = 0.079, Fig. 6b), indicating that changes in the gut microbiota of *R. dybowskii* did not alter bacterially mediated physiological functions. Significant differences were observed in six Kyoto Encyclopedia of Genes and Genomes (KEGG) pathways (cancers – overview, circulatory system, environmental adaptation, excretory system, infectious diseases – bacterial, and substance dependence) among the C, G, and R groups (Kruskal-Wallis H test, *P* < 0.05; Figure S8).

## Discussion

### Variation in gut microbiota diversity

Many studies have been carried out to examine the influence of antibiotics on the gut microbiota [4, 8]. However, these studies mainly used oral antibiotics, and few studies have examined the effects of bath-applied antibiotics on the microbiota of the interior of the body (such as the intestine). The present study showed that the ACE, Chao1, and Shannon indices of the gut microbiota in the G group were lower than those in the C group, and Venn diagrams illustrated that the number of unique OTUs was lower in the G group than in the R and C groups. These results are consistent with the microbiota of weaned piglets treated with chlortetracycline [23]. Antibiotic treatment caused changes in alpha diversity in individual honeybee hosts [10]. Similarly, an investigation of mosquito-eating fish exposed to antibiotics in water for 7 days showed that antibiotics significantly reduced the diversity of the gut and skin bacterial communities in the fish [24]. High microbiota diversity is favourable for the fitness and overall health of animals [22]. Many studies have shown that a reduction in bacterial diversity, the introduction of new species, and the total eradication of existing species are manifestations of the impact of antibiotics on the human and animal gut microbiota [8]. The use of antibiotics significantly reduces the alpha diversity of the gut bacterial community, which may be a manifestation of either the adverse side effects of antibiotics or dysbiosis of the gut bacterial community [25].

The ability of the gut microbiota to recover to the baseline level after antibiotic treatment is stopped may vary depending on differences in antibiotic administration, host species, community context, and environmental reservoirs [3, 26]. In the present study, the gentamicin bath significantly changed the composition of the gut bacterial community; after 7 days, the gut microbiota was still much as it had been during the gentamicin bath. After mice were treated with antibiotics, it was found that the structure of the microbiota in the intestines of the mice was significantly changed but could be quickly restored to its previous state after treatment [3]. However, in previous studies that examined antibiotic treatment in aquatic animals, the intestinal microbiota biodiversity was reduced within a few days of antibiotic consumption, and the initial composition of the bacterial community rarely fully recovered [27]. In an animal model of mosquito-eating fish exposed to water containing antibiotics, the diversity of the gut microbiota of the fish rapidly decreased, and the composition of the microbiota changed [24]. The effect of antibiotics on the gut bacterial community persists after the withdrawal of antibiotic treatment. Severe antibiotic pressure results in irreversible, long-lasting alterations in the gut microbiota [8, 10, 28, 29].

### Variation in the gut microbiota composition

Some studies involving humans or animals have shown that the use of antibiotics has a significant effect on the relative abundances of some bacteria in the gut bacterial community [6]; usually, the less abundant phylum Proteobacteria is enriched, while the relative abundance of Bacteroides and Firmicutes decreases [2, 3]. However, in this study, no significant differences were found in the abundance of bacterial phyla among the C group, the G group, and the R group. This may be due to the use of antibiotic baths rather than internal administration; bath application prevents the drugs from entering the intestinal tract directly and thus limits the impact on the gut microbiota.

In the present study, LEfSe analysis indicated that *Morganella* was significantly enriched in the G group (LDA > 4, *P* < 0.05). The composition of the gut bacterial community of *R. dybowskii* before and after antibiotic treatment and the differences in microbial profiles between these groups may be related to the selective pressure of gentamicin. Aside from pathogens that may have a strong tolerance to gentamicin, the concentration of the remaining bacteria in the treatment group may be related to the severe stress imposed by gentamicin. Organisms such as *Morganella* are functional bacteria associated with the formation of bacterial biofilms [30], and the bacteria in such biofilms are strongly resistant to antibiotics; the formation of biofilms is a fundamental cause of resistance in bacteria [29, 31]. When bacteria are in an environment that is not conducive to their growth, they form a mutually adherent bacterial community to resist the action of antibiotics. These reasons may explain the change in the composition of the gut bacterial community of *R. dybowskii* under pressure from gentamicin.

### Frogs in an antimicrobial bath

Oral and topical medications are preferred for animal use because they are safe, rapid, and reliable [31]. The antibiotic gentamicin may penetrate the surface of the skin, the largest and most accessible organ of the animal body, and travel through its layers to reach the circulatory system, which may lead to changes in the gut microbiota, e.g., by disrupting the steady state of the gut microbiota. In this study, gentamicin was used at a dose of 20 mg/L, which is similar to the doses of gentamicin and other antibiotics administered to frog through medicated baths in previous studies [11, 16, 31]. The drug bath method is non-invasive and causes less stress to animals than other methods, possibly reducing animal pain and damage to the skin [11, 16, 31]. Although a few reports have discussed the treatment of frog diseases with medicinal baths, most medicines are recommended for oral use or through injection [7, 11]. This lack of information indicates that dermal treatments for frog use are often chosen based on the treatment efficacy in other species and that the dose is inferred from its use in these species. Riviere et al. [16] found that gentamicin can enter the bloodstream after a gentamicin bath, but when the concentration of gentamicin in the bath was increased (from 10 to 50 µg/mL), the concentration of gentamicin in the blood increased only slightly (from 1.4 µg/mL to 1.5 µg/mL). This finding indicates that the relationship between the concentration of the drug in the blood and the concentration of the drug in the bath may be complicated. However, in this study, we did not determine the concentration of the drug in the blood or use a gradient of different drug concentrations. Further research is needed to obtain a deeper understanding of the relationship between the amphibian gut microbiota and antibiotics.

The capacity to absorb a drug may vary across animals and skin types. In the same species, the ability to absorb drugs through the skin may vary across different areas of the body [14]. The drug absorption capacity of the same skin area can vary widely across different host species and is often associated with the habitat of the host species [14]. For example, aquatic frogs tend to have a relatively uniform skin thickness, with modest vascularization in all skin areas, while the ventral skin of terrestrial or tree-dwelling species tends to be much thinner and more vascularized than the dorsal skin. In this study, the depth of the bath was 1.8 cm, which ensured that the liquid could soak one-third of the body height of each brown frog. The abdomen of each frog was mostly immersed in the liquid, which may have promoted percutaneous absorption of the drug [16]. The medicinal bath could affect the diversity of the skin bacterial community, and it is well known that the skin bacterial community plays a vital role in amphibian health. The bacterial community of amphibian skin can vary with the external environment, which may lead to changes in the species and abundance of opportunistic pathogens and endanger the fitness and health of the host [12]. The skin microbiota is particularly important for maintaining the health of amphibians, more so than in other animals. Antibiotic baths may disturb the stability of the skin microbiota; thus, it is necessary to consider this issue when treating amphibian diseases through antibiotic baths.

The gut microbiota is easily disturbed by internal and external factors [32]. Among humans and livestock, antibiotic exposure is a key source of interference that can severely alter the gut microbiota composition [33]. Few studies have examined the effects of antibiotic baths on the gut microbiota. Antibiotics may be used to treat humans or animals when they develop a disease; however, antibiotic abuse can cause much harm [10]. The steady state of the gut bacterial community is crucial; if the steady state is disrupted, the pathogens or potential pathogens in the intestine have the opportunity to multiply in large numbers and break through the intestinal mucosa into the tissue, eventually leading to systemic infection [28]. In the present study, the relative abundance levels of some potential pathogenic bacteria were significantly reduced, and the functional prediction of infectious diseases, such as those caused by bacteria and environmental adaptation, was increased in the drug bath group, which may represent a positive effect of the antibiotics [34].

### Effect of an antimicrobial bath on the function of the gut microbiota

Functions are sometimes conserved among diverse microorganisms, and functional redundancy may occur in the microbiota [21]. In this study, there was no significant and positive association between the composition and functional composition of the gut microbiota between the G and C frogs, indicating that the functional redundancy of the gut bacterial community was high. However, studies have shown marked differences in the gut microbiota between healthy and diseased animals (e.g., *R. dybowskii*), and a significant positive correlation exists between the community and functional composition, indicating that the composition of the gut bacterial community in diseased animals has reduced functional redundancy [22]. The degree of gut dysbiosis caused by antibiotics and diarrhoea may differ. Diarrhoea is also probably followed by the disappearance of functional redundancy, which may be related to the severity and progression of dysbiosis [18]. The functional redundancy of the same species may differ among developmental states [18]. For example, gut redundancy may be much higher in infants than in adults [18].

## Conclusions

In this study, we dissected the impact of antibiotics on potential pathogenic bacteria and gut microbiota diversity in *R. dybowskii* and examined the correlation between the structure of the intestinal bacterial community and its predicted functional components. Gentamicin significantly changed the structure of the gut microbiota, and the microbiota exhibited weak resilience, failing to recover after seven days. A few potentially pathogenic bacteria associated with red-leg syndrome were significantly depleted after the gentamicin bath. The gentamicin bath did not change the functional composition of the gut microbiota of *R. dybowskii*, and there was no significant correlation between the taxonomic composition of the gut microbiota and the functional composition, illustrating the high functional redundancy of the frog gut bacterial community. These findings provide insight into the role of the safe use of antibiotics in amphibians and the alleviation of the effects of antibiotic treatment on the gut microbiota.

## Methods

### Sample collection

The 21 *R. dybowskii* used in this study were caught in August 2017 on a farm in Huanan County (N 46°44′54″, E 130°69’32″, 80 m alt.), Heilongjiang, China. The captive *Rana dybowskii* were cultivated in a greenhouse, where sparse vegetation was planted, water sprayers and shade nets were installed, and the ground humidity was 25–35 %. The frogs were fed yellow mealworms (*Tenebrio molitor*) at 3 % of their body mass twice per day. The culture density was approximately 40/m^2^. The frogs on the farm were not diagnosed with any disease and were not treated with antibiotics. During the experiment, the frogs were collected from the farm, transported to the laboratory, and kept in a laboratory terrarium for 5 days. The three groups of frogs were raised in separate plastic boxes (43.0 × 32.0 × 27.7 cm^3^) in the laboratory. Each plastic box was covered at the bottom with a watery pad, which guaranteed that the frog skin was wet [12]. During the experiment, the frogs were raised at 16 °C, and the frogs were fed live mealworms (brought from the farm along with the frogs) at a rate of 3 % of their body mass once a day.

Twenty-one *R. dybowskii* (18.07 ± 0.39 g) individuals were separated into the G group, R group, and C group (*n* = 7 each, male-to-female ratio of 3:4). The frogs in the G group were subjected to a gentamicin (E003632; Sigma, US) bath at 20 mg/L for 60 min every day for a week. The liquid in the antibiotic bath was configured every day to ensure the concentration of antibiotics. Simultaneously, the C group was treated with a distilled water bath for 7 days. The R group was maintained for another week after receiving the same treatment as the first group. The bath depth was 1.8 cm, which ensured that the liquid could soak one-third of the body height of each brown frog. The depth of the treatment bath was set according to the size of the frogs to promote the percutaneous absorption of the drug [16]. Previous studies showed that frogs soaked in 50 µg/mL solution had plasma gentamicin concentrations up to 1.5 µg/mL [16].

In this study, gut and skin samples were collected after the frogs were euthanized according to previous studies [3]. Within 20 min after euthanasia, the frogs were dissected to expose the intestines, and the gut contents were sampled. The digestive tract of each frog was cautiously isolated, and the intestine from the pylorus to the anus was obtained. A fresh pair of sterile tweezers was used for each frog to avoid cross-contamination. The contents of each intestine were poured into a sterile vial and quickly stored at -80 °C.

All animal protocols were approved by the Institutional Animal Care and Use Committee of Northeast Agricultural University (IACUC#2015-035). All experiments were performed according to the approved guidelines and regulations. All experiments involving animals followed the principles of the 3 Rs (replacement, reduction, and refinement) to prevent unnecessary killing [35].

### DNA extraction and PCR amplification

Genomic DNA was extracted with a FastDNA® Spin Kit for Soil (MP Biomedicals, US) according to the manufacturer’s instructions. Using a GeneAmp 9700 PCR thermocycler (ABI, US), the hypervariable V3–V4 region of the bacterial 16 S rRNA gene in each specimen was amplified with the primers 5′-ACTCCTACGGGAGGCAGCAG-3′ and 5′-GGACTACHVGGGTWTCTAAT-3′. The PCR program was as follows: initial denaturation at 95 °C for 3 min; 27 cycles of denaturation at 95 °C for 0.5 min, elongation at 55 °C for 0.5 min, and elongation at 72 °C for 0.75 min; and extension at 72 °C for 10 min. The PCRs were repeated three times using the following 20-µL system: 5× buffer (4 µL), polymerase (0.4 µL) (both FastPfu), 5 µM primers (0.8 µL each), template DNA (10 ng), 2.5 mM deoxynucleotide triphosphates (dNTPs, 2 µL each), and BSA (0.2 µL). The PCR products were separated using electrophoresis with a 2 % agarose gel, purified with an AxyPrep DNA Gel Extraction Kit (Axygen Biosciences, US), and quantified using QuantiFluor™-ST (Promega, US) as instructed by the manufacturers.

### Illumina MiSeq sequencing

The pure amplicons were pooled in equimolar concentrations and subjected to paired-end (2 × 300) sequencing on a MiSeq system (Illumina, US) according to the manufacturer’s instructions. Raw FASTQ files were sent for de-multiplexing, quality C via Trimmomatic and integration via Fast Length Adjustment of Short reads (FLASH) with three criteria: (1) the primers were exact matches with no more than two nucleotide mismatches, and reads with ambiguous bases were discarded; (2) sequences with more than 10 bp of overlap were integrated per overlap; and (3) reads with a mean quality score <20 over a 50-bp sliding window at any site were deleted [36].

The OTUs were clustered using UPARSE (http://drive5.com/uparse/) at a 97 % similarity limit, and chimaeric sequences were identified and removed using UCHIME [37]. The taxonomic assignment of each 16 S rRNA gene sequence was performed by RDP Classifier 2.2 (http://sourceforge.net/projects/rdp-classifier/) with reference to the relevant SILVA database (Release119, www.arb-silva.de) at a confidence limit of 70 % [38].

### Ecological and statistical analyses

The software mothur 1.30.2 (https://www.mothur.org/wiki/Download_mothur) was used to generate rarefaction curves and calculate alpha diversity indices [39]. Differences in the alpha diversity indices (ACE, Chao1, Sobs, and Shannon) were analysed via the Wilcoxon rank-sum test. The core OTUs were those that existed in all samples from each group and represented ≥0.1 % of the reads. To evaluate the differences in the gut microbiota among the different groups, we calculated the Bray-Curtis dissimilarities and unweighted UniFrac similarity values on an out-level table using analysis of similarities (ANOSIM) and the Adonis multivariate ANOVA procedure [40]. The data were visualized through non-metric multidimensional scaling (NMDS). The unique and shared OTUs were represented in a Venn diagram generated using R software 3.0.0 (R Core Team, New Zealand) [41].

The relative abundances of the phyla and genera in the C, G, and R groups were statistically compared by the Kruskal–Wallis H test. Given that multiple tests were performed, the *P*-values were corrected for the false discovery rate (FDR), and the confidence interval (CI) was computed by Scheffer software. The relative abundances of the phyla in the microbiota in the two groups were determined via Welch’s t-test. Significant connections between bacterial taxa and host groups were identified by LEfSe [42], which considers both consistency and statistical significance and can recognize differentially abundant taxa among groups.

KEGG Orthology (KO) functional profiling was performed with Phylogenetic Investigation of Communities by Reconstruction of Unobserved States (PICRUSt) based on 16 S rRNA sequencing data [43]. Significant differences in the relative abundances of the predicted functions among the C, G, and R groups were evaluated by the Kruskal–Wallis H test. Only differences with *P-*values < 0.05 are presented.

General discrepancies in the phylogenetic structures and forecasted functional compositions were assessed by similarity analysis using the Bray-Curtis distance and by principal coordinate analysis (PCoA) for visualization, and the connections among the composition changes were examined using ANOSIM. The indicator taxa connected to each group were identified by the indicator value (IndVal) method [44]. The analyses were completed using the “labdsv” package in R v3.0.0. Rare taxa that could incorrectly reflect special taxa were rejected [45]. In this study, only taxa with IndVal > 0.90 (*P* <0.05) and relative abundance > 0.1 % were retained [44, 46].

### Potentially pathogenic genera

Several bacteria were chosen for comparison to clarify the potentially pathogenic bacteria causing red-leg syndrome in the gut of *R. dybowskii*. *Streptococcus*, *Staphylococcus*, *Pseudomonas*, *Proteus*, *Edwardsiella*, *Chryseobacterium*, *Citrobacter*, and *Aeromonas* are all potential pathogenic genera related to red-leg syndrome in amphibians [20, 47–50]. Differences in the potentially pathogenic genera were evaluated by the Wilcoxon rank-sum test.

## Supplementary Information

**Additional file 1 : Table S1.** Core OTUs among all samples. **Figure S1.** Rarefaction curves. (**a**) Sob curves and Shannon curves (**b**) of all samples. The Sob curves are plots of the number of OTUs as a function of the number of sequences. The Shannon curves reflect the microdiversity of the samples. **Figure S2.** The Venn diagram. The Venn diagram shows the shared communities among the control (C), gentamicin (G), and recovery (R) groups at the OTU level. **Figure S3.** The numbers of core OTUs and shared OTUs. The curve shows the core OTUs for all samples (a). The curve shows that the numbers of core OTUs in the control (C), gentamicin (G) and recovery (R) groups decreased as the sample size increased. The Venn diagram shows the shared OTUs among the C, G, and R groups at the OTU level (b). **Figure S4.** Bacterial community composition of different samples at the phylum level. Only genera with relative abundances over 1% in at least one sample are shown here. **Figure S5.** Microbial genes with a significant difference in different groups*.* a: *Morganella*, b: *Weissella*, c: *unclassified_f__Veillonellaceae*, d: *Crenobacter*, and e: *unclassified_f__Eggerthellaceae*. X-axis: the relative abundance of bacterial genera in each group. Microbial genes with a significant difference between the control, gentamicin, and recovery groups by Kruskal–Wallis H test*.* The difference between proportions (%) is displayed within the set confidence interval. *: 0.01 < *P* ≤ 0.05; **: 0.001 < *P* ≤ 0.01. **Figure S6.** Heatmap of the top 35 dominant bacteria at the genus level with respect to abundance. Cluster analysis was performed using Bray-Curtis distances and the average-linkage method. Each bar or column corresponds to a specimen. The data are calculated in terms of relative abundance, and the colours represent log values. **Figure S7.** The relative abundance of potentially pathogenic genera changed after antibiotic baths. The relative abundance of potentially pathogenic genera changed after antibiotic baths by Kruskal-Wallis H test (0.01 < *P* ≤0.05 marked as *, *P* ≤0.01 marked as **). **Figure S8.** The relative abundance of predicted genes in the metagenome. The left list represents KEGG pathways at level 1, the middle list represents KEGG pathways at level 2, and the heatmap represents the abundance of each functional pathway for each sample. Asterisks indicate significant differences among the groups (*P* < 0.05).

## Data Availability

All sequence data have been deposited in the NCBI Sequence Read Archive (SRA) under the BioProject accession numbers PRJNA635022 (https://www.ncbi.nlm.nih.gov/bioproject/PRJNA635022), PRJNA626516 (https://www.ncbi.nlm.nih.gov/bioproject/PRJNA626516), and PRJNA691728 (https://www.ncbi.nlm.nih.gov/bioproject/PRJNA691728).
