# Supplementary material for: Changes in the gut microbiota diversity of brown frogs (Rana dybowskii) after an antibiotic bath
Source: BMC Vet Res. 2021 Oct 21;17:333. doi: 10.1186/s12917-021-03044-z (PMC8529755; doi:10.1186/s12917-021-03044-z)
Supplement: Supplementary file 1 — Additional file 1 : Table S1. Core OTUs among all samples. Figure S1. Rarefaction curves. (a) Sob curves and Shannon curves (b) of all samples. The Sob curves are plots of the number of OTUs as a function of the number of sequences. The Shannon curves reflect the microdiversity of the samples. Figure S2. The Venn diagram. The Venn diagram shows the shared communities among the control (C), gentamicin (G), and recovery (R) groups at the OTU level. Figure S3. The numbers of core OTUs and shared OTUs. The curve shows the core OTUs for all samples (a). The curve shows that the numbers of core OTUs in the control (C), gentamicin (G) and recovery (R) groups decreased as the sample size increased. The Venn diagram shows the shared OTUs among the C, G, and R groups at the OTU level (b). Figure S4. Bacterial community composition of different samples at the phylum level. Only genera with relative abundances over 1% in at least one sample are shown here. Figure S5. Microbial genes with a significant difference in different groups. a: Morganella, b: Weissella, c: unclassified_f__Veillonellaceae, d: Crenobacter, and e: unclassified_f__Eggerthellaceae. X-axis: the relative abundance of bacterial genera in each group. Microbial genes with a significant difference between the control, gentamicin, and recovery groups by Kruskal–Wallis H test. The difference between proportions (%) is displayed within the set confidence interval. *: 0.01 < P ≤ 0.05; **: 0.001 < P ≤ 0.01. Figure S6. Heatmap of the top 35 dominant bacteria at the genus level with respect to abundance. Cluster analysis was performed using Bray-Curtis distances and the average-linkage method. Each bar or column corresponds to a specimen. The data are calculated in terms of relative abundance, and the colours represent log values. Figure S7. The relative abundance of potentially pathogenic genera changed after antibiotic baths. The relative abundance of potentially pathogenic genera changed after antibiotic baths by Krus [file 12917_2021_3044_MOESM1_ESM.docx]

**Supplementary materials**

**Changes in the gut microbiota diversity of brown frogs (*Rana dybowskii*) after** **an antibiotic bath**

Qing Tong^a,b,c^, Li-Yong Cui^b^, Jia Bie^a^, Xiao-Yun Han^c^, Zong-Fu Hu^a^, Hong-Bin Wang^a^, Jian-Tao Zhang^a*^

^a^College of Veterinary Medicine, Northeast Agricultural University, Harbin, 150030, China

^b^Jiamusi Branch of Heilongjiang Academy of Forestry Sciences, Jiamusi, 154002, China

^c^College of Life Science, Jiamusi University, Jiamusi, 154007, China

***Corresponding author**

Jian-Tao Zhang

College of Veterinary Medicine, Northeast Agricultural University, Harbin, 150030, People's Republic of China

E-mail: zhangjiantao@neau.edu.cn;

Tel.: +86-451-55190470

**Table S1**. Core OTUs among all samples.

| Phylum | Genus | OTU | C-Mean | C-Sd | G-Mean | G-Sd | R-Mean | R-Sd |
| --- | --- | --- | --- | --- | --- | --- | --- | --- |
| Bacteroidetes | *Bacteroides* | OTU417 | 3.23% | 3.99% | 3.18% | 3.92% | 3.92% | 3.65% |
| Bacteroidetes | *Bacteroides* | OTU364 | 0.73% | 1.36% | 0.99% | 1.32% | 0.73% | 0.49% |
| Bacteroidetes | *Bacteroides* | OTU38 | 3.37% | 2.99% | 2.04% | 1.21% | 1.72% | 1.12% |
| Bacteroidetes | *Bacteroides* | OTU402 | 0.12% | 0.15% | 0.09% | 0.06% | 0.09% | 0.06% |
| Bacteroidetes | *Bacteroides* | OTU14 | 0.40% | 0.27% | 0.30% | 0.15% | 0.31% | 0.12% |
| Bacteroidetes | *Bacteroides* | OTU728 | 0.10% | 0.09% | 0.09% | 0.08% | 0.07% | 0.03% |
| Bacteroidetes | *Bacteroides* | OTU466 | 0.37% | 0.71% | 0.48% | 0.70% | 0.64% | 0.65% |
| Bacteroidetes | *Alistipes* | OTU416 | 1.04% | 1.94% | 1.42% | 1.87% | 1.07% | 0.68% |
| Firmicutes | *Faecalitalea* | OTU333 | 3.54% | 6.35% | 4.85% | 6.07% | 6.33% | 5.37% |
| Firmicutes | *Vagococcus* | OTU198 | 8.68% | 15.59% | 11.61% | 15.10% | 13.89% | 14.34% |
| Actinobacteria | *Rhodococcus* | OTU448 | 0.10% | 0.11% | 0.12% | 0.10% | 0.14% | 0.09% |
| Actinobacteria | *Microbacterium* | OTU165 | 0.16% | 0.22% | 0.21% | 0.20% | 0.20% | 0.16% |
| Actinobacteria | *Arthrobacter* | OTU753 | 2.35% | 3.48% | 3.08% | 3.26% | 3.44% | 3.07% |
| Proteobacteria | *Citrobacter* | OTU179 | 7.39% | 5.72% | 6.66% | 5.28% | 5.81% | 4.60% |
| Proteobacteria | *Acinetobacter* | OTU41 | 1.04% | 2.22% | 1.45% | 2.18% | 1.04% | 0.88% |
| Proteobacteria | *Morganella* | OTU470 | 1.10% | 1.00% | 1.18% | 0.92% | 1.14% | 0.67% |


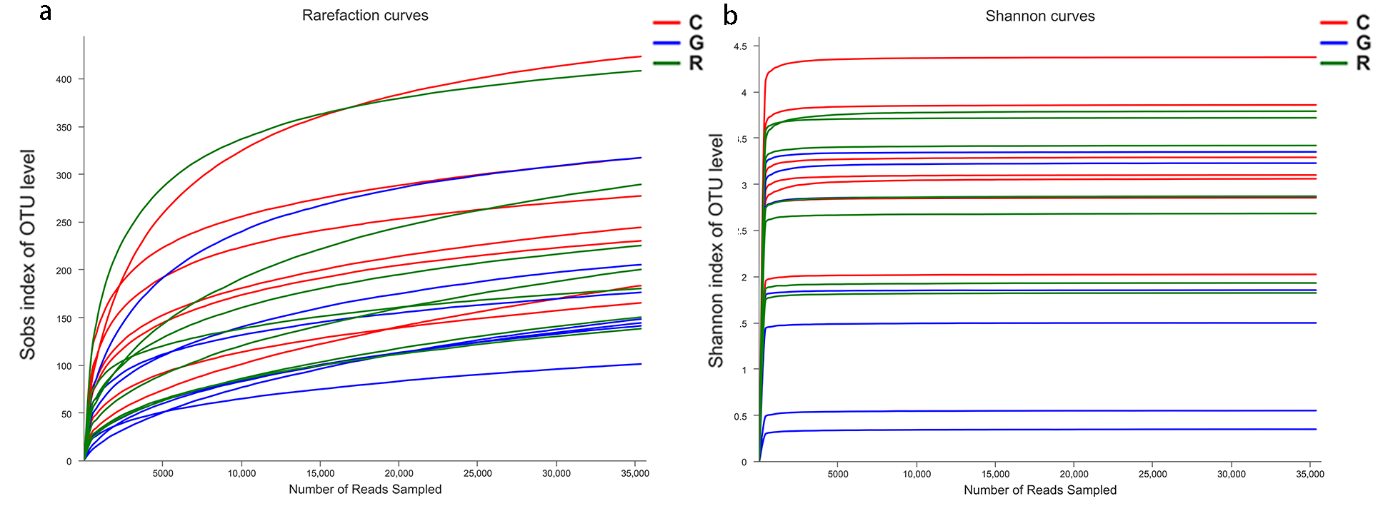
 **Figure S1.** Rarefaction curves.

(**a**) Sob curves and Shannon curves (**b**) of all samples. The Sob curves are plots of the number of OTUs as a function of the number of sequences. The Shannon curves reflect the microdiversity of the samples.

**
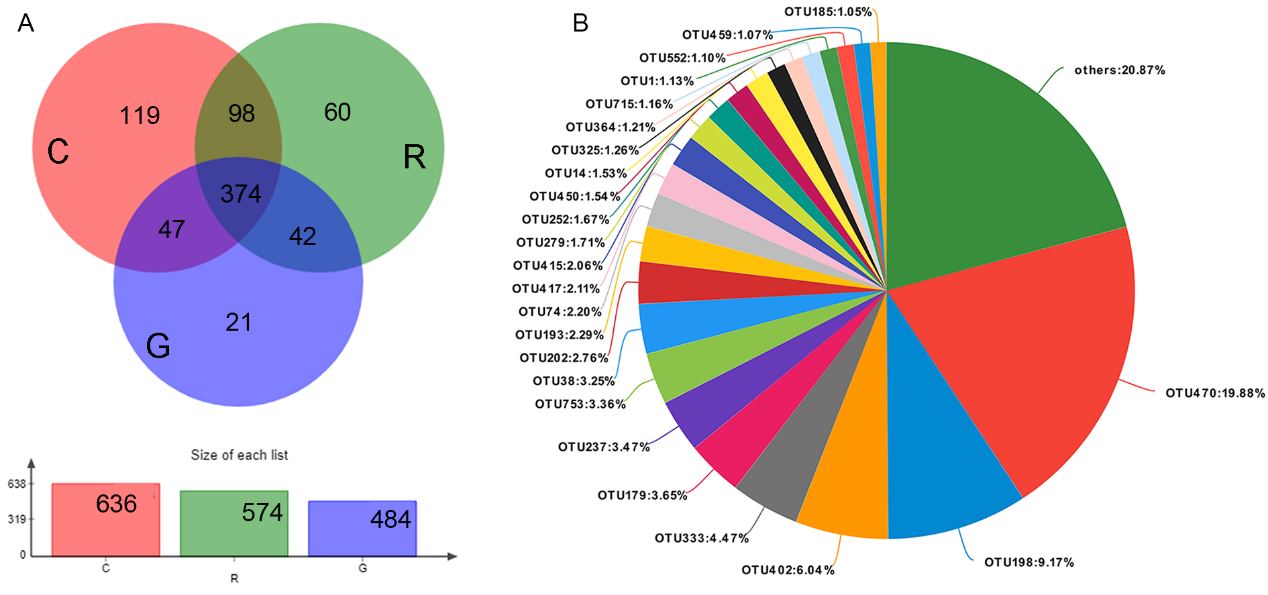
**

**Figure S2.** The Venn diagram.

The Venn diagram shows the shared communities among the control (C), gentamicin (G), and recovery (R) groups at the OTU level.


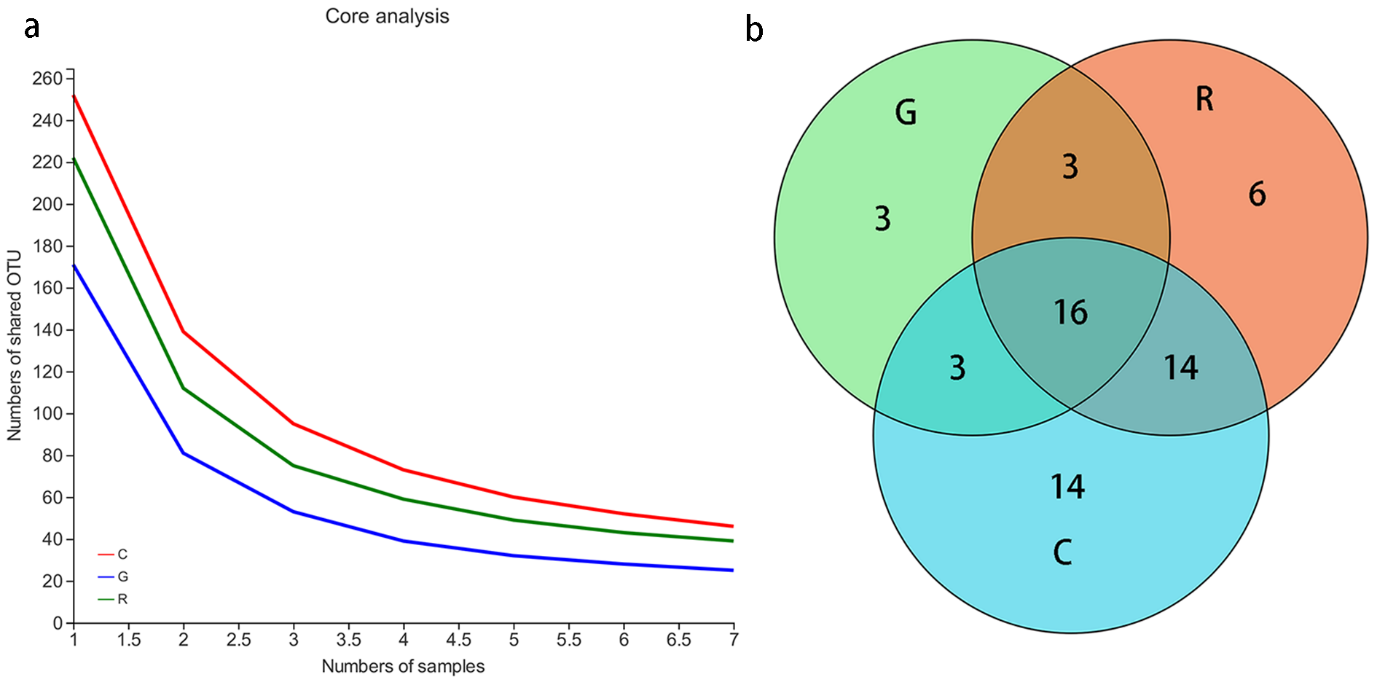


**Figure S3.** The numbers of core OTUs and shared OTUs.

The curve shows the core OTUs for all samples (a). The curve shows that the numbers of core OTUs in the control (C), gentamicin (G) and recovery (R) groups decreased as the sample size increased. The Venn diagram shows the shared OTUs among the C, G, and R groups at the OTU level (b).

**
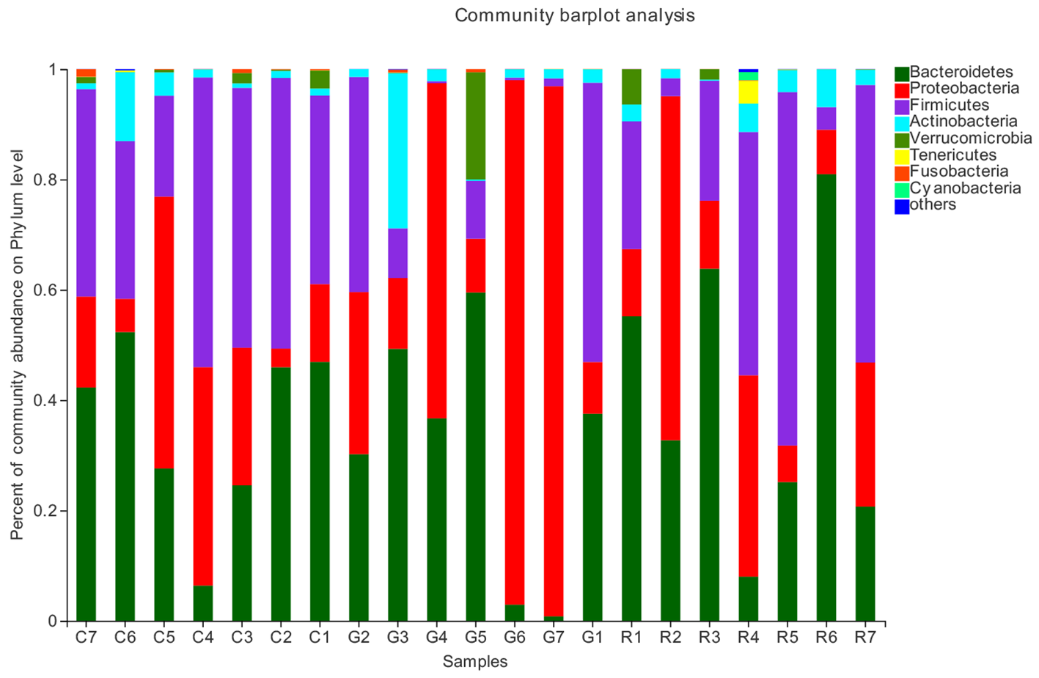
**

**Figure S4.** Bacterial community composition of different samples at the phylum level.

Only genera with relative abundances over 1% in at least one sample are shown here.


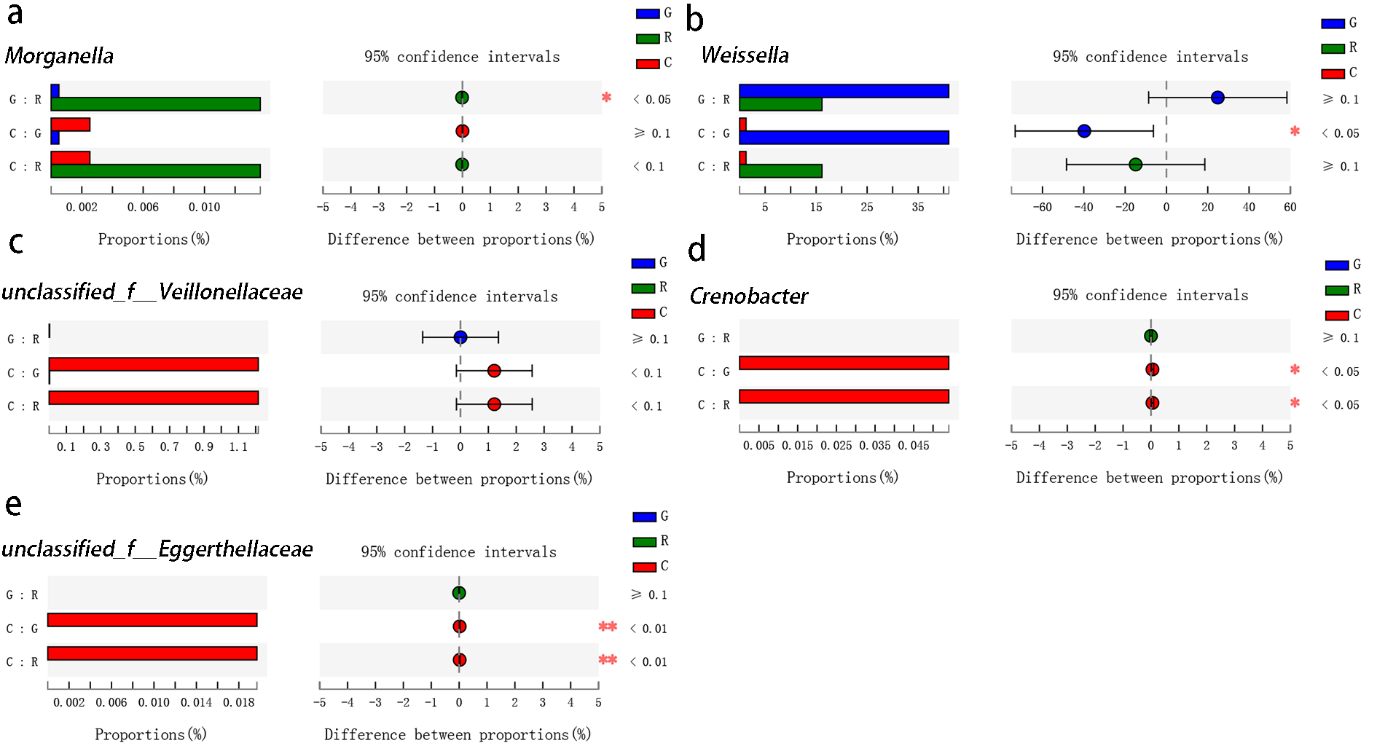


**Figure S5.** Microbial genes with a significant difference in different groups*.*

a: *Morganella*, b: *Weissella*, c: *unclassified_f__Veillonellaceae*, d: *Crenobacter*, and e: *unclassified_f__Eggerthellaceae*. X-axis: the relative abundance of bacterial genera in each group. Microbial genes with a significant difference between the control, gentamicin, and recovery groups by Kruskal–Wallis H test*.* The difference between proportions (%) is displayed within the set confidence interval. *: 0.01 < *P* ≤ 0.05; **: 0.001 < *P* ≤ 0.01.

**
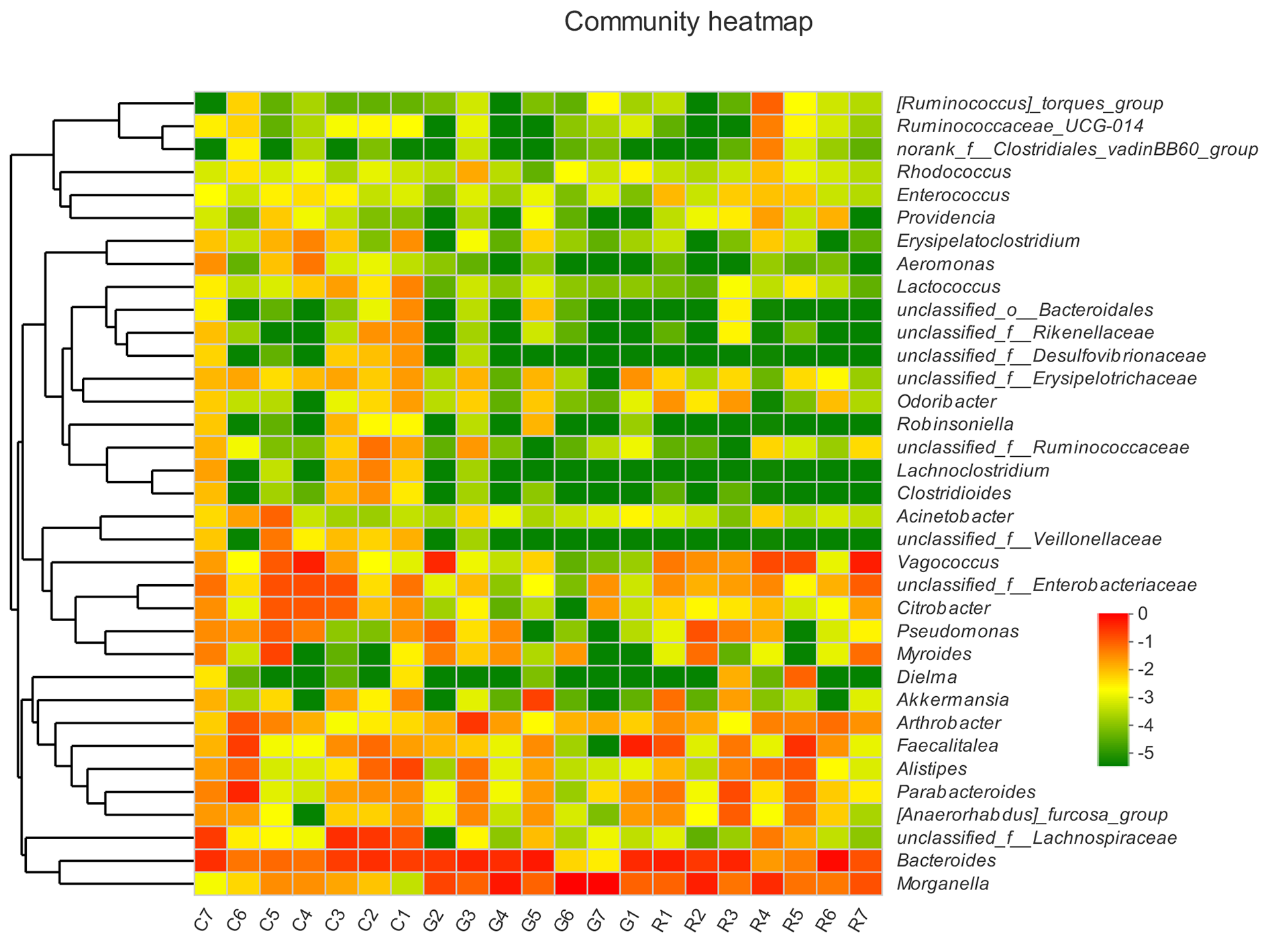
**

**Figure S6.** Heatmap of the top 35 dominant bacteria at the genus level with respect to abundance.

Cluster analysis was performed using Bray-Curtis distances and the average-linkage method. Each bar or column corresponds to a specimen. The data are calculated in terms of relative abundance, and the colours represent log values.


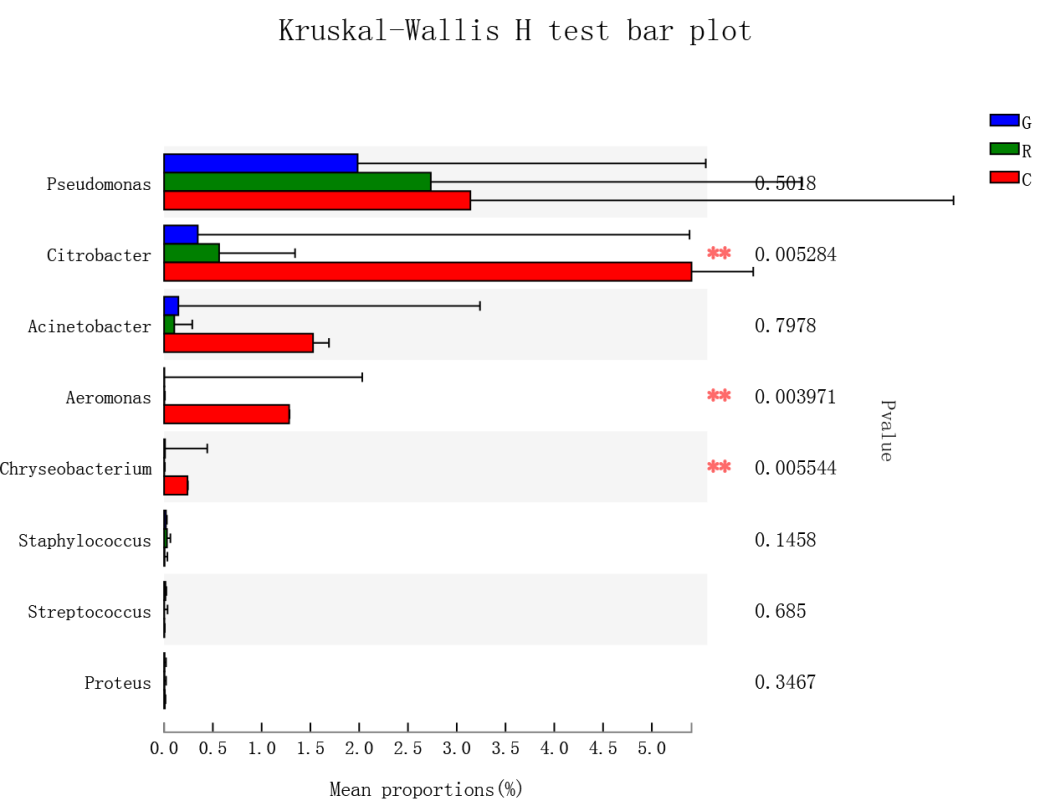


**Figure S7.** The relative abundance of potentially pathogenic genera changed after antibiotic baths.

The relative abundance of potentially pathogenic genera changed after antibiotic baths by Kruskal-Wallis H test (0.01 < *P* ≤0.05 marked as *, *P* ≤0.01 marked as **).


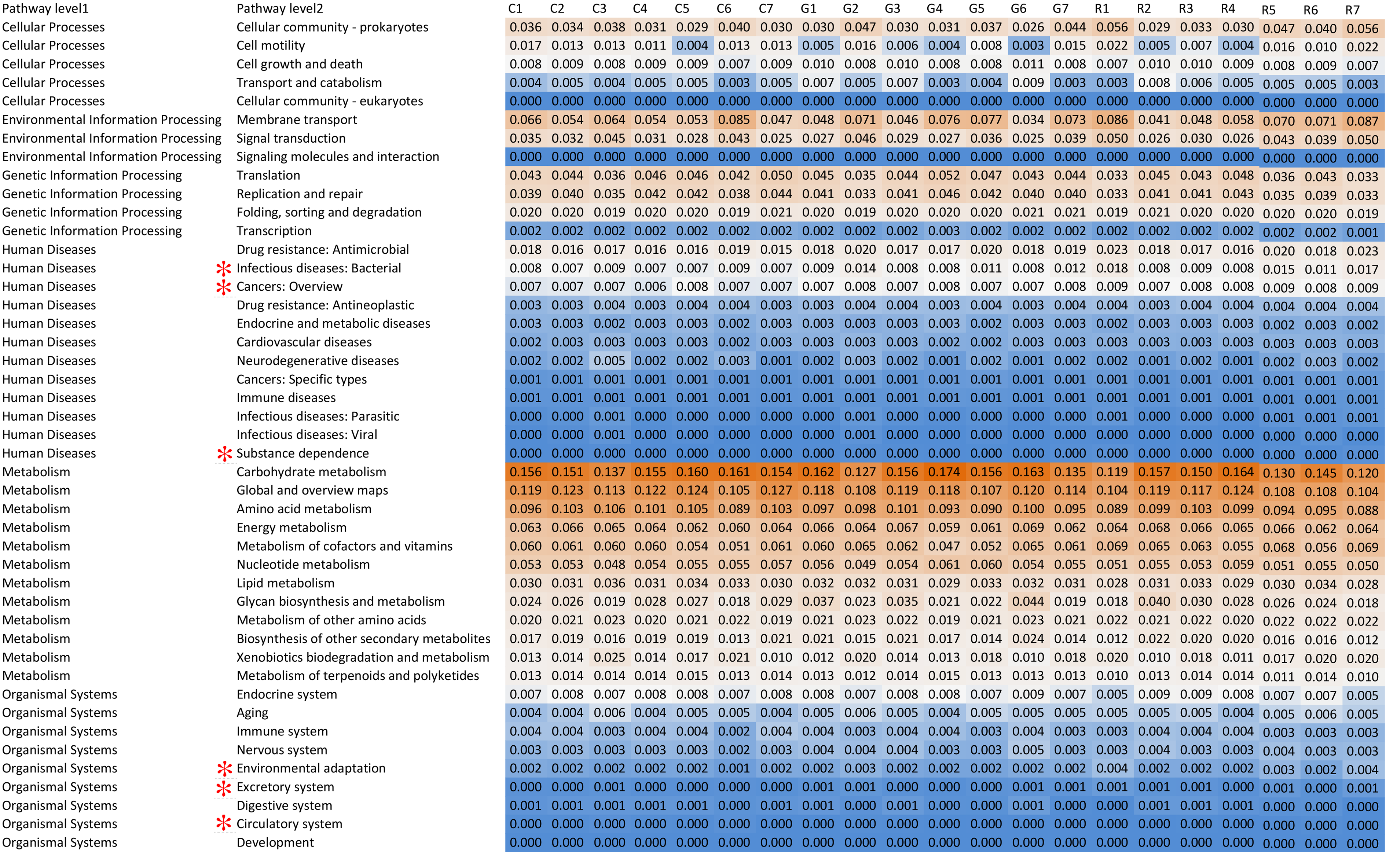


**Figure S8.** The relative abundance of predicted genes in the metagenome.

The left list represents KEGG pathways at level 1, the middle list represents KEGG pathways at level 2, and the heatmap represents the abundance of each functional pathway for each sample. Asterisks indicate significant differences among the groups (*P* < 0.05).
